# Tumor-Associated Macrophage (TAM) and Angiogenesis in Human Colon Carcinoma

**DOI:** 10.3889/oamjms.2015.044

**Published:** 2015-04-27

**Authors:** Manal A. Badawi, Dalia M. Abouelfadl, Sonia L. El-Sharkawy, Wafaa E. Abd El-Aal, Naglaa F. Abbas

**Affiliations:** *Pathology Department, Medical Division, National Research Centre, Dokki, Giza, Egypt*

**Keywords:** Adenomatous polyps, cancer colon, macrophages, angiogenesis, CD68, Factor VIII

## Abstract

**AIM:**

This study aimed to clarify how macrophages affect prognosis in cancer colon and their association with tumor angiogenesis.

**MATERIAL AND METHODS:**

Forty four biopsies of colon carcinoma and 15 of benign adenomatous polyps were investigated for macrophages infiltration and microvessels density using immunohistochemistry and image morphometric analysis. Macrophages and blood vessels were stained immunohistochemically by CD68 and F-VIII markers respectively. The morphometric analysis was carried out on the immunohistochemically stained slides using the Leica Qwin 500 Image Analyzer. Both of macrophages infiltration and microvessels density were correlated with histological tumor grade, stage and lymph node metastases and were correlated with each others.

**RESULTS:**

Macrophage infiltration was significantly higher in malignant cases than in benign polyps. High macrophage infiltration and hypervascularity were significantly correlated with T-staging and lymph nodes metastasis. A significant correlation was found between macrophage infiltration and microvessels densitie in malignant tumors where hypervascularity was significantly correlated with high macrophages infiltration.

**CONCLUSION:**

The significant correlation between macrophage infiltration and tumor angiogenesis suggests an interaction between macrophages and cancer cells stimulating microvessels formation, tumor invasion and metastasis in colon cancer. We recommend that macrophages infiltration should be evaluated to investigate their clinical value in development of individualized therapeutic regimens for management of colon carcinoma.

## Introduction

The tumor microenvironment encompasses a wide variety of cells including malignant and non-malignant populations. Non-malignant populations include stromal cells, an expanding vasculature, and a leukocyte infiltrate. Macrophages comprise the dominant portion of the leukocyte population [1].

Tumor-associated macrophages (TAMs), macrophages existing tumor microenvironment, were first described in the early 1980s [2, 3]. Till now, TAMs have been found to play dual functions, both positively or negatively affect tumor growth through interactions with the micro-environment, and these actions are tissue specific [4-6].

Macrophages exist in two distinct polarized states: one is the classically activated (M1) state and the other is the alternatively activated (M2) state. M1 macrophages possess antitumor activity, whereas M2 macrophages promote tumor invasion and metastasis [7, 8]. However, most TAMs have a M2-like phenotype. CD68 is a pan-macrophage marker frequently used as a marker for TAMs, and CD68 recognizes both tumoricidal M1 and anti-inflammatory M2 macrophages [9, 10].

The vast majority of studies with numerous tumor types, including follicular lymphoma [11], gastric carcinoma [12], pancreatic cancer [13] and thyroid carcinoma [14] show that the presence of TAM in the tumor microenvironment is associated with a worse prognosis, some studies claim the opposite [15]. The specific role of TAMs in colon cancer is more controversial [16].

Cancer development and progression is a complex multistep process where novel capabilities, the hallmarks of cancer, are acquired through the accumulation of multiple genetic alternations. The ability to activate angiogenesis plays a crucial role in controlling tumor progression because tumor growth, invasion, and metastasis are angiogenesis dependent [17], In addition, angiogenesis facilitates metastasis. The newly formed tumor blood vessels are structurally abnormal. For instance, increased numbers of fenestrations, vesicles, and vesicovacuolar channels and a lack of normal basement membrane are common in tumor vessels [18]. These abnormal blood vessels are consequently more permeative and would constitute the easier entry point for tumor cells to enter into the circulation and hence distant micrometastases [19, 20].

Tumor angiogenesis is crucial for tumor progression. Tumor angiogenesis was initially thought to be induced only by tumor cells themselves; however, tumor-associated macrophages are indeed a major player in the regulation of tumor angiogenesis [21].

This study aimed to clarify how macrophages affect prognosis in cancer colon and their association with tumor angiogenesis.

## Material and Methods

Forty four specimens of colon carcinoma and 15 of benign adenomatous polyps were included in this study. The specimens were selected randomly from the Pathology Department, Faculty of Medicine, Cairo University. Sections of 5 micrometer thickness were prepared, stained for H&E, CD68 and factor VIII. The pathology reports and hematoxylin and eosin stain slides were revised and tumor grading was performed according to WHO classification. The presence of lymph node metastasis and T-stage were reviewed. Ten fields from each slide were investigated for macrophages infiltration and microvessels density using immunohistochemistry and image morphometric analysis.

### Immunohistochemical study

CD68 and factor VIII expression was examined in all tissues using streptavidin-biotin technique. Two slides from each case were deparaffinized, hydrated and incubated in 3% hydrogen peroxide for 30 minutes to block the internal peroxidase activity. Antigen retrieval was done by microwave pretreatment for 10 minutes in 0.01M citrate buffer. For each case, one slide was incubated at 4ºC overnight with monoclonal antibodies against CD68 (Medicopharma trade) with a dilution 1:200. The second slide was incubated with monoclonal antibodies to factor VIII at a dilution 1:500 (Dako Corporation). These steps were followed by 30 minutes incubation with biotinylated horse anti-mouse antibody at room temperature, avidin-biotin peroxidase complex for 50 minutes at room temperature and finally diamiobenzidine (DAB) for 3-5 minutes. The slides were counter stained with hematoxylin, dehydrated and mounted.

CD68 expression was scored as the percentage of positively stained cells of stromal/inflammatory cells as follow: score I, up to 20%; score II, 20-50%; score III, > 50% [22].

In the negative control group, 1% bovine serum albumin was used in place of the primary antibody.

### The morphometric analysis

The morphometric analysis was carried out on the immunohistochemical stained slides using the Leica Qwin 500 Image Analyzer (LEICA Imaging Systems Ltd, Cambridge, England) at the Pathology Department, National Research Center. We place the slide to be examined on the stage of the microscope, and focus it at power magnification (100X) for counting blood vessel density and (200X) for counting macrophage infiltration. The light source is set to the required level. Successful adjustment of illumination is checked for on the video monitor.

Macrophage infiltration was evaluated by calculating the percentage of CD68 positively stained cells of stromal/inflammatory cells in 10 fields (x 200).

Microvessls density was evalulated by calculating the mean number of microvessels in 10 fields (x 100). Cases with mean number 140 or less were considered as hypovascular tumors and cases with mean number more than 140 were considered as hypervascular tumors [23].

Both of macrophage infiltration and vascular density were correlated with histologic grades, T-staging and lymph nodes status. Also they were correlated with each other.

### Statistical Analysis

Chi-square (χ²) test was applied to examine the correlation between macrophage infiltration, vascular densities and histologic grade, lymph node metastases, and tumor tissue invasion (T-staging). P-value less than 0.01was considered significant.

## Results

Immunostaining for CD68 and Factor VIII was evaluated in 15 benign adenomatous polyps and 44 cases of colon carcinoma. According to WHO, 10 cases were GI, 18 cases of GII and 16 cases of GIII. On reviewing T-staging of the cases, 8 cases were T2, 26 cases T3 and 10 cases were T4. Of all cases of colon carcinoma, 36 cases showed lymph nodes metastasis (N1, N2) and 8 cases were lymph nodes negative (N0).

### Macrophages infiltration

CD68-positive macrophages were located in the stroma of adenomatous polyps, while in colon carcinoma, they were in the stroma and in particular along the tumor front.

**Figure 1:**
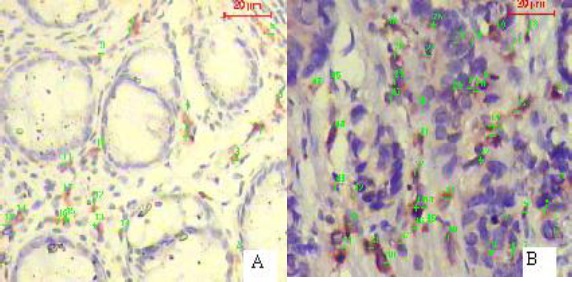
*Immunohistochemical expression of CD68, macrophage specific marker, in adenomatous polyp (A) and colon carcinoma (B). Note that colon carcinoma is more infiltrated by macrophage than adenomatous polyp (Immunohistochemistry x 200)*.

Macrophage infiltration was significantly higher in malignant cases than in benign polyps (p < 0.01) where (45.5%) of colon carcinoma and 20% of adenomatous polyps showed score III macrophages infiltration. On the contrary, 80% of adenomatous polyps and 47.7% of carcinoma cases revealed score I macrophages infiltration (Figure 1).

Pathological parameters were compared in tumors with different macrophage scores. There was a non-significant (p > 0.01) correlation between macrophage infiltration and the histologic grade. On the other hand, macrophage infiltration was significantly correlated with (p < 0.01) T-staging and lymph nodes metastasis (Table 1) where high macrophage infiltration score was more frequently detected with high T-stages and lymph nodes metastasis.

**Table 1:** Correlation between macrophages infiltration and clinicopathological parameters in colon carcinoma.

	No.	Macrophage infiltration score		
		I	II	III
**Grade**				
I	10	6 (60%)	2 (20%)	2 (20%)
II	18	10 (55.5%)	5 (27.8%)	3 (16.7%)
III	16	5 (31.25%)	2 (12.5%)	9 (56.25%)
**Stage**				
T2	8	6 (75%)	2(25%)	-
T3	26	14 (53.9%)	5 (19.2%)	7 (26.9%)
T4	10	1 (10%)	2 (20%)	7 (70%)
**L.N.**				
N0	10	8 (80%)	2 (20%)	-
N1	18	8 (44.4%)	5 (27.8%)	5 (27.8%)
N2	16	5 (31.25%)	2 (12.5%)	9 (56.25%)
**Total**	44	21 (47.7%)	9 (20.5%)	14 (31.8%)

### Microvessels density

Microvessels density was significantly higher (p < 0.01) in malignant cases than colonic polyps, where the mean microvessels count was 130 and 64 in both groups respectively.

Of the 15 colonic polyps, 14 cases (93.3%) were hypovascular and the remaining 2 cases (6.7%) were hypervascular.

**Figure 2:**
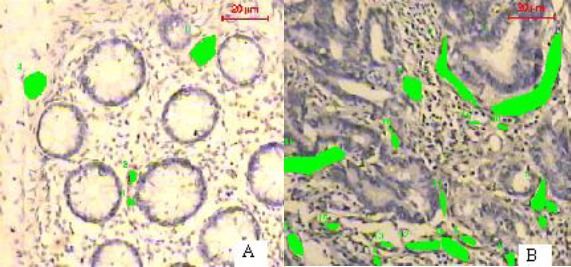
*Adenomatous polyp (A) and colon carcinoma (B) immunohistochemically stained for FVIII revealing more vascularity of carcinoma than adenomatous polyp (Immunohistochemistry x 200)*.

In malignant cases, hypervascularity was detected in 2l cases (47.7%), while the remaining 23 cases (52.3%) were hypovascular (Figure 2).

Histologic grade was not significantly different (p > 0.01) between hypovascular and hypervascular tumors. On the contrary, hypervascularity was significantly (p < 0.01) correlated with high T-stages and lymph nodes metastasis (Table 2).

**Table 2:** Correlation between microvessels density and clinicopathological parameters in colon carcinoma.

	No.	Hypervascular	Hypovascular
**Grade**			
I	10	3 (30%)	7 (70%)
II	18	9 (50%)	9 (50 %)
III	16	9 (56.3)	7 (43.7%)
**Stage**			
T2	8	2 (25%)	6 (75%)
T3	26	12 (46.2%)	14 (53.8%)
T4	10	7 (70%)	3 (30%)
**L.N.**			
N0	8	2 (25%)	6 (75%)
N1	17	8 (47.1%)	9 (52.9%)
N2	19	11 (57.9%)	8 (43.7%)
**Total**			
	44	21 (477%)	23 (52.3%)

### Correlation between macrophages and microvessels densitie

A significant correlation (p < 0.01) was found between macrophage infiltration and microvessels density in malignant tumors where hypervascularity was significantly correlated with high macrophages infiltration (Table 3).

**Table 3:** Correlation between macrophage infiltration and microvessels density in cancer colon.

	No.	Macrophage infiltration		
		I	II	III
Hypovascular	23 (52.3%)	15 (65.3%)	5 (21.7%)	3 (13%)
Hypervascular	21 (47.7%)	6 (28.6%)	4 (19%)	11 (52.4)
Total	44	21 (47.7%)	9 (20.5%)	14 (31.8%)

## Discussion

Cancer progression is a complex multi-step process that consists of transformation, tumor growth, invasion and metastasis. Tumor invasion and metastasis are the critical steps in determining the aggressive phenotype of human cancers [24]. The interaction between cancer cells and the stromal cells has been shown to promote cancer metastasis. The macrophages within the tumor, referring to as tumor-associated macrophages (TAMs), are the pivotal member of stromal cells and they are one of the predominant tumor-infiltrating immune cells supporting key processes in tumor progression [25, 26].

This study aimed to clarify how macrophages affect prognosis in cancer colon and their association with tumor angiogenesis.

There have been numerous studies regarding the role of TAMs in malignant progression, yet, in general, there is no clear consensus regarding the functional significance of TAMs in solid tumors and their metastasis [25, 27].

Inflammatory pathways can promote an environment which favors tumor growth and metastasis. Several infections, inducing inflammation, have been directly linked to cancer [15]. Chronic inflammation is implicated in the development of colorectal cancer, and the plasma inflammatory biomarker macrophage inhibitory cytokine-1 (MIC-1, GDF15) may have a direct role in tumorigenesis [28]. Macrophages are among the first cells to infiltrate infected or damaged tissue. Tumor-associated macrophages, which constitute a significant part of the tumor-infiltrating immune cells, have been linked to the growth, angiogenesis, and metastasis of a variety of cancers associating with worse prognosis [12-14, 29], other studies claim the opposite [15].

Inflammatory bowel disease like Crohn’s disease and ulcerative colitis (the pathophysiological mechanism of which are importantly driven by macrophages) are the real risk factors of colon cancer [30, 31]. Several lines of evidence showed that TAM can play an important role in the transformation of neoplasms [32]. For example, IL-1B secreted by activated macrophages has been shown to induce tumorigenic progression by increasing neovascu-larization [33], and TNF-alpha secreted by macrophages increased invasiveness of cancer cells via MMP-dependent mechanism [34]. So, Andrej Jedinak et al. [31] suggested that the inflammatory mediators (IL-1B, IL-6, and TNF- alpha) secreted by macrophages are responsible for the stimulation of proliferation and migration of colon cancer cells.

The results of the present study showed that cases of colon carcinoma have high macrophage infiltration compared to adenomatous colon polyps and macrophage infiltration significantly increase in parallel with clinical stage and lymph node metastasis not with histologic tumor grade. These results were in accordance with previous studies of Frank et al. [36]; Hagemann et al. [34] and Lin et al. [35]. In breast carcinoma also Medrek et al., [37] showed that TAMs (CD68 and CD163) may play important roles in carcinogenesis and tumor progression. He et al. [9] have clarified in their study on oral squamous cell carcinoma (OSCC) that positive expression of TAMs was significantly associated with aggressive behavior of the tumors including lymph node status. They added that these markers are associated with cancer stem cell markers and OSCC overall survival, suggesting their potential prognostic value in these tumors. On the other hand, other studies revealed that high macrophage infiltration in colon cancer correlated with better prognosis [38, 31]. It was suggested that the degree to which the antitumorigenic abilities may partly depend on macrophage contact with tumor cells, as well as to achieve a high macrophage to cancer cell ratio [38]. This indicates that the role of macrophages in colon cancer has not yet been clearly defined.

Angiogenesis is a tightly regulated event critical for tumor growth and is an essential component of tumor metastasis [39]. Tumor angiogenesis involves degradation of the basement membrane of the original vessel, endothelial cell activation, migration, proliferation and the formation of new capillaries. All these processes are controlled by angiogenic factors (e.g. VEGF) secreted either by the tumor cells or by the surrounding stroma [29]. Caffo et al. (40) study on gliomas have demonstrated that CD68- positive onoclonal cells were observed in perivascular and hypoxic areas isideband contiguous to vessel wall and suggested that macrophages could promote angiogenesis mechanisms and induction of tumor growth.

In the present study, microvesseles density was significantly higher in colon carcinoma than in colonic polyps. Also neovascularization was significantly correlated with high clinical carcinoma stages and lymph node metastasis, but not correlated with tumor histologic grades. These results were in accordance with Tarta study [41] which showed an association between microvessels density and deeper tissue invasion and a lower rate of survival and non-significant correlation with tumor histologic differentiation. On the contrary, Choi et al. [42] found a non-significant difference between high microvessel counts and deeper tumor invasion.

In the study of Andrej Jedinak et al. [31], it was found that conditioned media from activated macrophages stimulates HCT116 cells to secrete VEGF and further induce capillary morphogenesis of endothelial cells. These observations can be supported by the earlier studies showing that stimulation of cells with pro-inflammatory mediators (TNF-alpha, IL-B, and IL-6) increases the secretion of factors responsible for angiogenesis [36, 39]. These findings support our results where a significant correlation (p < 0.01) was found between macrophage infiltration and microvessels densities in malignant tumors and hypervascularity was significantly correlated with high macrophages infiltration.

In conclusion, the present study showed that macrophages infiltration is higher in malignant colon compared with benign adenoma. In addition the significant correlation between macrophage infiltration and tumor angiogenesis suggests an interaction between macrophages and cancer cells stimulating microvessels formation, tumor invasion and metastasis in colon cancer. We recommend that macrophages infiltration should be evaluated to investigate their clinical value in development of individualized therapeutic regimens for management of colon carcinoma.
